# Costs and cost-effectiveness of Shamiri, a brief, layperson-delivered intervention for Kenyan adolescents: a randomized controlled trial

**DOI:** 10.1186/s12913-023-09856-z

**Published:** 2023-08-04

**Authors:** Corinne N. Kacmarek, Natalie E. Johnson, Tom L. Osborn, Christine Wasanga, John R. Weisz, Brian T. Yates

**Affiliations:** 1 Department of Psychology, American University, 4400 Massachusetts Ave NW, Washington, DC 20016 USA; 2 Department of Clinical Research, Division of Clinical Epidemiology, University Hospital Basel, Totengässlein 3, 4051 Basel, Switzerland; 3 Shamiri Institute, 13th Floor, Pioneer Point (CMS Africa), Chania Avenue, Nairobi, Kenya; 4 Department of Psychology, Kenyatta University, Box 43844, Nairobi, 00100 Kenya; 5 Department of Psychology, Harvard University, 1030 William James Hall, 33 Kirkland Street, Cambridge, MA 02138 USA; 6 Department of Psychology, American University, 4400 Massachusetts Ave NW, Asbury Building Room 321, Washington, DC, 20016-8062 USA

**Keywords:** Cost-effectiveness, Global mental health, Adolescents, Lay provider, Depression, Anxiety, School-based, Health promotion, Low and middle income country

## Abstract

**Background:**

Low- and middle-income countries (LMICs) have the highest socio-economic burden of mental health disorders, yet the fewest resources for treatment. Recently, many intervention strategies, including the use of brief, scalable interventions, have emerged as ways of reducing the mental health treatment gap in LMICs. But how do decision makers prioritize and optimize the allocation of limited resources? One approach is through the evaluation of delivery costs alongside intervention effectiveness of various types of interventions. Here, we evaluate the cost-effectiveness of *Shamiri*, a group– and school–based intervention for adolescent depression and anxiety that is delivered by lay providers and that teaches growth mindset, gratitude, and value affirmation.

**Methods:**

We estimated the cost-effectiveness of *Shamiri* using the Consolidated Health Economic Evaluation Reporting Standards (CHEERS) guidelines for economic evaluations. Changes in depression and anxiety were estimated using the Patient Health Questionnaire (PHQ-8) and Generalized Anxiety Disorder questionnaire (GAD-7) at treatment termination and 7-month follow-up using two definitions of treatment benefit. Cost-effectiveness metrics included effectiveness-cost ratios and cost per number needed to treat.

**Results:**

Base case cost assumptions estimated that delivering *Shamiri* cost $15.17 (in 2021 U.S. dollars) per student. A sensitivity analysis, which varied cost and clinical change definitions, estimated it cost between $48.28 and $172.72 to help 1 student in *Shamiri*, relative to the control, achieve reliable and clinically significant change in depression and anxiety by 7-month follow-up.

**Conclusions:**

*Shamiri* appears to be a low-cost intervention that can produce clinically meaningful reductions in depression and anxiety. Lay providers can deliver effective treatment for a fraction of the training time that is required to become a licensed mental health provider (10 days vs. multiple years), which is a strength from an economic perspective. Additionally, *Shamiri* produced reliable and clinically significant reductions in depression and anxiety after only four weekly sessions instead of the traditional 12–16 weekly sessions necessary for gold-standard cognitive behavioral therapy. The school setting, group format, and economic context of a LMIC influenced the cost per student; however, broader conclusions about the cost-effectiveness of *Shamiri* have yet to be determined due to limited economic evaluations of mental health programs in LMICs.

**Trial registration:**

This study was registered prior to participant enrollment in the Pan-African Clinical Trials Registry (PACTR201906525818462), registered 20 Jun 2019, https://pactr.samrc.ac.za/Search.aspx.

## Contributions to the literature

- Analyzing intervention costs, alongside effectiveness, in a low- and middle-income country (LMIC) fills an important gap in the global mental health literature.
- Shamiri can be delivered for a cost of $15.17 2021 U.S. dollars per student, which is acceptable in LMICs with purchasing power that is similar to Kenya’s.
- Cost savings come from use of paraprofessionals (i.e., lay providers), integration in a LMIC, integration in a school setting, and group-based format.
- *Shamiri* shows promise as a cost-effective, scalable mental health intervention that can expand access to mental health treatment for adolescents in LMICs.

## Background

Mental health concerns are a leading cause of disability worldwide, as well as one of the largest sources of economic burden [1]. This burden of mental disorders is especially high among youth in low- and middle-income countries (LMICs) [2]. As such, developing and disseminating evidence-based interventions for youth mental health has emerged as an urgent global health priority over the last decade [3]. Even though there has been increased research dedicated to developing and testing the efficacy of youth mental health interventions in LMICs, very few of these attempts have included economic evaluations of these interventions. Indeed, such economic evaluations of evidence-based interventions are extremely rare globally. One recent systematic review of economic evaluations revealed only one evaluation of an intervention for youth depression, and zero for anxiety [4].

How then do decision makers choose which interventions to prioritize in LMICs where chronic government under-investment in mental healthcare persists [5, 6]? Economic evaluations of youth mental interventions, in addition to efficacy examinations, are needed to inform important policy, practice, and research decisions by policymakers, practitioners, researchers, and other mental health professionals interested in investing limited resources wisely. It seems that efforts to expand help-seeking options for youth mental health in LMICs are handicapped insofar as research efforts emphasize efficacy evaluation without robust economic evaluations.

One approach to reducing the youth mental health treatment gap in LMICs involves the development of brief and scalable youth mental health interventions. The work on brief and scalable interventions is inspired by three very promising ideas: First, simple intervention strategies that focus on improving broader human functioning, rather the reduction of psychopathology, and cultivating individual character strengths can be effective for many youth mental health problems [7–9]. These interventions, which are sometimes called “wise” interventions or “character strength” interventions [10], tend to focus on single and simple human attributes, like “growth mindset”, and show promise as effective interventions for youth mental health problems [11]. Second, task-shifting to lay providers with minimal to no formal mental health training can be an effective avenue for expanding the presently limited mental health caregiving workforce. Lay providers have been shown to be capable of effectively delivering a wide array of mental health interventions across diverse settings in LMICs [12, 13]. Task-shifting has indeed emerged as a WHO-recommended approach to mental health caregiving in LMICs [14]. Finally, a community-orientation to help–seeking can be effective for tackling societal stigma, getting buy–in, and mobilizing existing resources and infrastructure for mental healthcare [15, 16]. Indeed, community–delivered interventions are often found to be feasible, acceptable, and effective for youth mental health problems in LMICs [17].

The *Shamiri* (Kiswahili for “thrive”) intervention, a brief group-based intervention for adolescent depression and anxiety symptoms, was developed using these three ideas [18]. First, the intervention consists of three simple intervention strategies drawn from the “wise” intervention literature: (1) growth mindset, which teaches youth that their personal attributes and characteristics are malleable and can change through effort, (2) gratitude, which encourages them to notice and appreciate things in their lives, and (3) value affirmations, which teaches them to take value-aligned actions [18, 19]. Second, the intervention is delivered by lay providers aged 18 to 22 after only 10 hours of training [18–20]. Third, the intervention is group-based and is delivered in schools, i.e., a community-based setting, as an afterschool program. It is delivered in only four one hour sessions across four weeks [21]. The efficacy of the Shamiri intervention has been tested in at least two randomized controlled trials (RCTs) including in a recent RCT which found that youth assigned to the *Shamiri* condition experienced greater reductions in symptoms of depression and anxiety than those assigned to an active study-skills control condition [18, 21].

The RCTs revealed that *Shamiri’s* efficacy was similar to those of traditional evidence-based youth mental health interventions, but *Shamiri* differed in its *low-intensity low-touch* intervention approach. Most evidence-based interventions for youth mental health are resource intensive: they are delivered by expert caregivers (with a master’s or doctoral-level background), use a one-on-one format, are implemented in office settings, and last 12–16 weeks [22]. It seems that *Shamiri’s* low-intensity approach (use of lay providers, community–based delivery, and brevity) may yield significant cost-savings compared to traditional delivery systems. An economic evaluation of the *Shamiri* intervention can allow us to test this premise. Thus, we conducted an economic evaluation to investigate whether *Shamiri* is a cost-effective way to promote mental health for youths in Kenya. Assessing the cost-effectiveness of *Shamiri* can facilitate its comparison with other mental health interventions that have been subject to economic evaluation. Comparisons of that kind could guide such policy decisions as which mental health programs to invest in, in LMICs [23]. In line with best practices [4, 24–26], costs were estimated from multiple perspectives, and sensitivity analyses were performed to address sources of uncertainty regarding clinically meaningful improvements.

## Methods

### Trial design, treatment conditions and outcomes

We used data from a recent pre-registered RCT of *Shamiri* [20, 21]. Participants (*n* = 413) were Kenyan high school students with elevated self-reported symptoms of depression or anxiety. Students were aged 13 to 18 and attended four public secondary schools in Nairobi and Kiambu County. Students were randomly assigned to *Shamiri*, a four-week intervention delivered by a lay provider, or to an active study skills control condition. The study skills control condition was also delivered by lay providers and was designed to match the *Shamiri* condition in terms of duration and structure. This design allowed for *Shamiri* to be evaluated against a rigorous active control which provides the highest benchmark for efficacy [22]. Students in both conditions met in groups of 7–15 students. Each group had four weekly meetings lasting one hour. Study procedures were approved by the Maseno University Ethics Review Committee and the National Commission for Science, Technology, and Innovation.

The lay provider training was created and conducted by our study team. It consisted of one hour of introduction and overview, one hour on the study rules and expectations, two hours on peer counseling skills and emergency protocol, and six hours of content role plays. As part of the emergency protocol, lay providers were supervised by students with counseling experience, and trained to elevate any risks that arose during sessions to their supervisors. The supervisors consulted PhD-level clinical and counseling psychologists in the case of medium and high risk cases [27].

Outcomes were collected at baseline, midpoint, post-treatment, 2-week follow-up, and 7-month follow-up. Primary outcomes were self-reported depressive symptoms and anxiety symptoms. Depressive symptoms were assessed using the *Patient Health Questionnaire-8* (PHQ-8), which removes the suicidal ideation item from the PHQ-9 because it is considered stigmatizing to Kenyan adolescents [28] Anxiety symptoms were assessed using the *Generalized Anxiety Disorder-7-item screener* (GAD-7) [29]. Both measures have demonstrated strong psychometric properties in samples of Kenyan adolescents [18, 21, 30]. To be eligible for the trial, students were required to have a score of  ≥ 15 on the PHQ-8 or  ≥ 10 on the GAD-7 [21]. Additional details about recruitment, lay provider training, and the intervention can be found in earlier publications [20, 21].

### Cost assessment

We used Yates’ (1997) [31] resources → procedures → processes → outcomes model to estimate the costs of the lay provider intervention from multiple perspectives. This approach begins by estimating the monetary value (i.e., cost) of *resources* necessary to implement the intervention’s *procedures*. First, we used a micro-costing approach to estimate the cost of intervention resources. This approach identifies the resources required for intervention activities by multiplying the amount of each resource used, such as hours in treatment, by the local cost of a unit of those resources, such as $20 per hour. We used an opportunity value approach [24, 32] to estimate unit costs. All costs were reported in 2021 U.S. dollars (USD). Given the uncertainty inherent in unit cost estimation, we calculated costs under three different scenarios. The base case scenario used our best estimate of the amounts and unit costs of each resource used to deliver the intervention in the present study. The low- and high-cost scenarios examined how modifications to the amount of each resource used would decrease or increase the total intervention cost per student. In the present study, we followed the Consolidated Health Economic Evaluation Reporting Standards (CHEERS) reporting guidelines [33].

### Effectiveness analyses

In our trial of *Shamiri*, we analyzed the impact of the intervention relative to the control group by applying mixed-effects linear models in which outcomes were entered as continuous variables [21]. In cost-effectiveness analyses, however, it is often useful to operationalize outcomes categorically in order to represent the cost per “clinically meaningful” improvement [34, 35]. Although these categorical analyses are useful for policymakers and health economists, there are also limits to analyzing mental health outcomes dichotomously [36]; thus, we also present effectiveness-cost ratios in which symptoms are treated continuously.

To define clinically meaningful improvement in depressive symptoms, we consulted previous literature on the PHQ-9 [37]. We applied two definitions of clinically meaningful change; first, we applied the “standard definition.” Under this definition, clinically meaningful improvement occurs if an individual a) starts with a score ≥ 10 and ends with a score ≤ 9, and b) experiences a reduction of at least 50% of their pre-treatment score [37]. Second, we applied the reliable and clinically significant change (RCSC) criterion C. Under this definition, clinically meaningful improvement occurs if an individual a) starts with a score ≥ 10 and ends with a score ≤ 9, and b) experiences a reduction of at least 5 (one standard deviation in clinical sample) points [37, 38]. Because the GAD-7 and PHQ-8 have the same cutoff scores for defining “minimal,” “mild,” “moderate,” and “moderately severe or severe” cases of depression and anxiety [28, 29], we applied the same criteria for defining clinically meaningful improvements in anxiety symptoms using the GAD-7 [29, 39].

The RCSC criteria is a superior measure of effectiveness compared to the standard definition because it has a greater empirical basis [38] and allows comparisons of treatment effectiveness across studies [37]. The 50% improvement needed for the standard definition is largely arbitrary [37]. However, the standard definition of improvement has shown good agreement (kappa > 0.6) with the RCSC definition for PHQ-9 depression [37]. Although the RCSC is considered a more valid indicator of clinical improvement, the standard definition of improvement has discriminated between treatment responders and non-responders for depression and anxiety symptoms [37, 39]. We include both definitions to represent diverse ways of classifying clinical change.

For each definition of clinically meaningful improvement, we calculated the *number needed to treat* (NNT) to result in a clinically meaningful improvement. The NNT provides an estimate of the number of people that need to receive *Shamiri* to result in one additional improvement, compared to what would be expected if everyone received the control condition [40].

### Effectiveness-cost analyses

We calculated the relationship between effectiveness and costs by calculating effectiveness-cost ratios (ECRs) for each student, which divides a student’s PHQ-8 change score or GAD-7 change score by the total cost per student. Effectiveness-cost ratios were preferred over cost-effectiveness ratios because the latter yield undefined quotients, which are then excluded from analyses, when the denominator is zero, i.e., when there is no change between a participant’s baseline and termination symptom scores [34]. We calculated the cost required to produce a clinically meaningful improvement by multiplying the cost per student by the NNT.

*Shamiri* and the active study skills control condition required the same amount of resources to deliver, and each was delivered in four sessions. Thus, treatment costs per student did not differ between conditions. As a result, some standard cost-effectiveness approaches (e.g., incremental cost-effectiveness ratios, two-part models) were not appropriate for our study. Instead, consistent with previous analyses in which costs did not differ between conditions [34] we focus on estimating costs, effectiveness-cost ratios, NNTs, and the cost per clinically meaningful improvement.

### Data analysis plan

Analyses were conducted in IBM SPSS Statistics for Mac, version 28 [41]. Missing data was handled using multiple imputation with seven datasets under the assumption that data were missing at random. Our primary effectiveness measure, incidence rates (for standard and RCSC definitions of clinically meaningful change), were calculated between baseline and treatment termination and baseline and 7-month follow-up. We report incidence rates that were pooled across the seven imputed datasets. Differences in effectiveness between conditions at both time points were evaluated using chi squared tests. The cost-effectiveness metric, cost per NNT, focused on data with statistically significant differences in effectiveness at either time point. Since SPSS could not produce pooled chi squared statistics and *p* values, effectiveness results were considered statistically significant if *p* values were less than 0.05 in at least four of the seven imputed datasets.

## Results

### Demographics

Our sample consisted of 413 students, 208 of whom were randomized to the control condition and 205 of whom were randomized to *Shamiri*. Average baseline GAD-7 scores for both groups were approximately 13, which suggests “moderate anxiety.” Both definitions of clinically meaningful improvement required baseline GAD-7 scores ≥ 10. Of 413 students, 94% reported baseline GAD-7 scores ≥ 10 (control = 197; *Shamiri* = 191). Average baseline PHQ-8 scores were also in the moderate range (12 for control and 13 for *Shamiri*), with 73% reporting PHQ-8 scores ≥ 10 (control = 147; *Shamiri* = 153). There were no statistically significant differences in baseline depression or anxiety symptoms between conditions [21]. A consort diagram is available in the parent trial (Osborn et al., [21], p. E5).

### Base case

#### Personnel costs

Lay provider, teacher, and supervisor time was necessary to deliver the intervention. Lay providers delivered the intervention, and teachers helped prepare students for the intervention. In addition, supervisors (four undergraduate students and one master’s student) met regularly with lay providers, and the supervisors met regularly with doctoral-level supervisors, to support intervention delivery. The monetary value of lay provider time (i.e., the unit cost), was estimated using the stipend lay providers received ($1.50/hour). In our estimate of total time devoted by lay providers, we included time delivering the intervention, in supervision, and traveling to and from the schools where they delivered the intervention and received supervision. Additionally, five supervisors provided support during sessions (e.g., providing lay providers with materials, navigating logistical challenges, supporting with time management). As part of their compensation package, lay providers received $2.99, per trip, for public transit passes to and from the schools; supervisors spent approximately $9.96, per trip, on transportation to and from the schools.

Because the intervention was conducted in a school, teachers spent approximately 10 minutes per session to transition students to intervention activities. Teacher time was estimated using the average monthly salary, including fringe benefits for teachers in Kenya ($4.16/hour). See Wasil et al., 2021 for additional details [34]. Additionally, two undergraduate supervisors met with lay providers twice a week, for a total of one hour per session, and provided administrative assistance during each session (e.g., gathering worksheets, keeping time). The undergraduate supervisors also met with a doctoral-level supervisor once per week for one hour during the four-week intervention.

We assumed that schools did not incur additional costs to acquire the facilities where the intervention was delivered. In other words, the school building was an *existing resource* that the intervention team had the privilege of using and did not need to pay for. Put another way, the cost of maintaining school facilities would be paid to a landlord by another party (i.e., school administrators) regardless of intervention delivery. Thus, our base case excludes the cost of acquiring and maintaining school facilities. Intervention delivery cost $6,267.22, or $15.17 per student. Base, low, and high estimates are presented in Table 1.

**Table 1:** Description of cost estimations per student in different scenarios

Budget Item	Unit Cost	Quantity	Unit	Base Case	Low Cost	High Cost
Teacher time	$4.16	2.72	hrs	$6.79. 10 mins per session, 4 teachers, 4 sessions/teacher		
Lay provider time	$1.50	168	hrs	$252.51. 3.5 intervention hours, 4 times, 12 lay providers		
Lay provider supervision	$1.50	60	hrs	$90.18. Four 30-min supervisory meetings with all lay providers. 15 min individual meetings with 12 lay providers		
Lay provider transit reimbursement	$2.99	12	trip	$35.88. Average round-trip costs per day, 12 lay providers	$17.94. Reduced by 50% from base case	$53.82. Increased by 50% from base case
Lay provider transit time	$1.50	825.6	hrs	$1,240.88. Unit cost for 1.15 hrs + 1 hr, 16 times^a^, twice per day (round trip), 12 lay providers	$620.44. Reduced by 50% from base case	$1,861.32. Increased by 50% from base case
Supervisor supervision	$19.13	116	hrs	$2,219.08. 2 undergraduate supervisors 1.75 hrs for 16 sessions^a^; 3 undergraduate supervisors for 1.25 hrs for 16 sessions^a^	$555.64. One undergraduate supervisor present at all sessions	$2,749.20. AB level qualification (GS-7 level with fringe benefits 2021)
Doctoral-level supervisor	$26.84	4	hrs	$107.34. Unit cost represents average hourly salary for 2 supervisors		
Supervisor transit reimbursement	$9.96	48	Trips/vehicle	$478.08. $9.96/trip, 1.5 cars, 16 episodes^a^, twice per episode (round trip)	$239.04. Reduced by 50% from base case	$717.12. Increased by 50% from base case
Supervisor transit time	$19.13^b^	96	hrs	$1,836.48. 1.2 hr round-trip travel time, 16 times, 5 supervisors	$918.24. Reduced by 50% from base case	$2,754.72. Increased by 50% from base case
Total costs				$6,267.22/413 students = $15.17/student	$2,808.12/413 students = $6.80/student	$8,593.00/413 students = $20.81/student

^a^ 4 schools × 4 sessions/school = 16. ^b^All supervisors were assigned GS-5 hourly rate, equivalent to a high school graduate

#### Recruitment, administrative and training costs

Four interviewers interviewed 18 candidates to serve as lay providers, each lasting 30 minutes, of which 12 were hired. The training lasted 10 hours across two days. Training costs included lay provider and trainer time spent in training, as well as traveling to and from training. A Kenyan-based site coordinator also spent approximately five hours per week, for four weeks, per school (80 hours total) coordinating intervention delivery. Since this coordinator had the same education level as the lay counselors, we assumed he was paid the same hourly rate. We are reporting implementation costs (recruitment, administrative, and training) and delivery costs separately. It is important to report implementation costs because they can help administrators decide whether they have the capacity to implement a new intervention. Adding such costs to delivery costs can inflate cost-outcome metrics since organizations differ in their existing resources and needs and, thus, will incur different implementation costs for the same intervention [42]. For example, a single school that is implementing *Shamiri* would not need a site coordinator. Implementation costs totaled $1,628.18 and are presented in Table 2.

**Table 2:** Description of recruitment, administration and training costs per student

Budget Item	Total Cost	Description
Trainer time	$382.60	10 hrs of training × 2 supervisors
Trainer transit time	$68.87	0.9 hrs × 2 days × 2 times per day (round trip)
Trainer transportation cost	$39.84	2 days of training × 2 times per day (round trip)
Lay provider training time	$15.03	$1.50 (unit cost), 10 hrs of training
Lay provider training transit time	$12.93	1.15 hrs at unit cost × 2 days × 2 times per day
Interviewer time	$688.68	4 interviewers × 18 candidates × 0.5 hrs /interview
Site coordinator	$120.24	5 hrs per week × 4 sites × 4 weeks
Other training costs	$300.00	Fixed cost per episode of training (2 days)
$1,628.18/413 students = $3.94/student		Total training and recruitment costs, and costs per student

## Low cost scenario

Our low cost scenario adjusted base case intervention assumptions in the following ways: All transportation costs were reduced by 50% and assumed that one (instead of five) undergraduate students led supervision. Varying some parameters by 50% to accommodate uncertainty has been reported in sensitivity analyses in other economic evaluations [43]. In our low cost scenario, it cost $6.80/student.

## High cost scenario

In our high cost scenario we increased base case transportation costs by 50%; we also assumed that the supervisors had a bachelor’s degree and were receiving fringe benefits. Using high cost assumptions, it cost $20.81/student.

## Effectiveness

### GAD-7

Relative to students in the control condition, students in the *Shamiri* condition experienced greater reductions in anxiety symptoms between baseline and treatment termination and between baseline and 7-month follow-up [21] (see Table 3). We also measured effectiveness dichotomously (met criteria vs. did not meet criteria) by calculating incidence rates for different definitions of clinical change across time, which can sometimes lead to conclusions that do not always align with those based on continuous measures of effectiveness.

**Table 3:** Mean GAD-7 and PHQ-8 scores by condition at multiple follow-ups

	Control *N* = 208			*Shamiri* *N* = 205		
	Baseline *M (SE)*	Termination *M (SE)*	7-month follow-up *M (SE)*	Baseline *M (SE)*	Termination *M (SE)*	7-month follow-up *M (SE)*
GAD-7	13.25 (0.24)	8.34 (0.40)	9.07 (0.61)	13.33 (0.24)	6.95 (0.35)	7.28 (0.45)
PHQ-8	12.41 (0.33)	8.34 (0.38)	9.73 (0.54)	12.91 (0.32)	7.20 (0.38)	8.01 (0.46)

At treatment termination, 63 of 208 (30%) students in the control condition and 39 of 205 (19%) students in *Shamiri* met the standard criteria for GAD-7 improvement. This difference was statistically significant or approached significance in all imputed datasets (*X*^*2*^ = 3.8 – 10.3, *ps* < 0.051). Also at treatment termination, 101 of 208 (49%) and 119 of 205 (58%) students in the control and *Shamiri* conditions, respectively, met the RCSC criteria, but this difference was not statistically significant in a majority of the imputed datasets (*p*s > 0.07). At 7-month follow-up, 45 of 208 (21%) and 38 of 205 (18%) students in the control and *Shamiri* conditions, respectively, met the standard criteria, but this difference was not statistically significant in a majority of the imputed datasets (*ps* > 0.09). However, the difference between the proportion of students who met the RCSC criteria for improvement at 7-month follow-up was statistically significant and favored *Shamiri* (110/205 = 54% vs. 88/208 = 42%; *X*^*2*^ = 4.9 – 9.0, *df* = 1, *ps* < 0.03*)*.

### PHQ-8

Relative to students in the control condition, students who received *Shamiri* experienced greater reductions in depression symptoms between baseline and treatment termination and baseline and 7-month follow-up (see Table 3 and Osborn et al., [30]). At treatment termination, 35 of 208 (17%) students in the control condition and 23 of 205 (11%) students in *Shamiri* met the standard criteria for PHQ-8 improvement; 81 of 208 (39%) and 97 of 205 (48%) met the RCSC criteria. These differences were not statistically significant in a majority of datasets (*ps* > 0.08 for standard criteria and > 0.07 for RCSC criteria). At the 7-month follow-up, 22 of 208 (11%) in the control condition and 27 of 205 (13%) in Shamiri met the standard criteria for PHQ-8 improvement (*ps* > 0.16). There was a statistically significant difference in the proportion of *Shamiri* (88/205 = 43%), relative to control (61/208 = 29%), participants who met RCSC improvement criteria, favoring *Shamiri*, in all of the imputed datasets (*X*^*2*^ = 4.2 – 14.1, *df* = 1, *ps* < 0.04*)*.

## Cost-effectiveness

### Effectiveness-cost ratios

Average GAD-7 ECRs for the control group at treatment termination ranged from 0.24 to 0.72, depending on the cost scenario. In other words, per dollar spent, there was an average 0.24 to 0.72-point reduction on the GAD-7 at treatment termination. Average ECRs for the *Shamiri* group ranged from 0.31 to 0.94 at treatment termination. Average GAD-7 ECRs were between 0.20 and 0.61 for the control group and between 0.29 and 0.89 for *Shamiri* at the 7-month follow-up. Average ECRs for the PHQ were similar to those for GAD-7: For the control group, they ranged from 0.20 to 0.60 at treatment termination and from 0.13 to 0.39 at 7-month follow-up. For *Shamiri*, they ranged from 0.27 and 0.84 at treatment termination and 0.24 and 0.72 at 7-month follow-up.

### Cost per number needed to treat

Costs per NNT for all measures, change definitions, and time points are in Table 4. In this text, we are only emphasizing cost per NNT for measures and time periods with statistically significant differences in incidence rates between conditions. At treatment termination a greater proportion of students in the control condition, relative to *Shamiri*, met the standard criteria for clinically meaningful GAD-7 improvement (30% vs. 19%); thus, 9.1 students would have to receive *Shamiri*, instead of the control, for one additional participant to *not* experience the standard GAD-7 reduction. The cost per NNT in the control condition, relative to *Shamiri,* would be between $61.88 and $189.37 for the different cost scenarios. At the 7-month follow-up, more students in *Shamiri,* relative to control, met the RCSC criteria for change (54% vs. 42%), making the NNT 8.3 and the cost per NNT in *Shamiri* between $56.44 and $172.72.

**Table 4:** Cost per multiple definitions of clinically meaningful improvement

Clinically meaningful improvement	Follow-up	Base	Low	High
Standard GAD-7	Termination	$138.05	$61.88	$189.37
	7-month	$505.16	$226.44	$692.97
RCSC GAD-7	Termination	$168.39	$75.48	$230.99
	7-month	$125.91	$56.44	$172.72
Standard PHQ-8	Termination	$253.34	$113.56	$347.53
	7-month	$758.50	$340.00	$1,040.50
RCSC PHQ-8	Termination	$168.39	$75.48	$230.99
	7-month	$107.71	$48.28	$147.75

At treatment termination, a greater proportion of students in the control condition, relative to *Shamiri*, met the standard criteria for clinically meaningful PHQ-8 improvement (17% vs. 11%); thus, 16.7 students would have to receive *Shamiri*, instead of the control, for one additional participant to *not* experience the standard PHQ-8 reduction. A greater proportion of students in *Shamiri*, relative to control, met the PHQ-8 RCSC criteria at termination (48% vs. 39%). For the RCSC criteria, 11.1 students would, on average, have to receive *Shamiri*, instead of control, for one additional patient to experience the RCSC PHQ-8 reduction. At the 7-month follow-up, more students in *Shamiri* met the RCSC criteria for PHQ-8 change (43% vs. 29%), making the NNT 7.1 and the cost per NNT between $48.28 and $147.75. Incidence and NNTs are summarized in Table 5.

**Table 5:** Number needed to treat at different definitions of clinical change and follow-ups

		Condition		
Clinically meaningful improvement	Follow-up	Control	*Shamiri*	Number needed to treat^a^
Standard GAD-7	Termination	30%	19%	9.1* ^b^
	7-month	21%	18%	33.3*
RCSC GAD-7	Termination	49%	58%	11.1*
	7-month	42%	54%	8.3^b^
Standard PHQ-8	Termination	17%	11%	16.7*
	7-month	11%	13%	50
RCSC PHQ-8	Termination	39%	48%	11.1
	7-month	29%	43%	7.1 ^b^

^a^The number needed to treat is the number of students who would have to receive Shamiri for one additional participant to have the specified outcome. When the incidence for the control condition is greater than Shamiri, it produces the number of people who would have to receive Shamiri to *not* have the specified outcome. These situations are represented with an asterisk. Numbers with a ^b^ were significant in a majority of the 7 imputed datasets

## Discussion

We aimed to identify the cost, effectiveness, and cost-effectiveness of *Shamiri*, a school-based intervention program delivered by lay providers in Kenya. At treatment termination, a greater proportion of students in the control condition reported reductions in anxiety according to a standard definition of change. Both definitions of clinically meaningful change have discriminated between treatment responders and non-responders for depression and anxiety symptoms, although some consider the RCSC a more valid indicator of clinical improvement [37, 39]. However, a greater percentage of *Shamiri* students, relative to students in a control group, experienced clinically significant reductions in depression and anxiety symptoms at a 7-month follow-up. Results of a sensitivity analysis suggest that it cost between $48.28 and $172.72 to help one student in *Shamiri*, relative to the control, achieve reliable and clinically significant change in depression and anxiety. Our study should be interpreted alongside its limitations. For example, we estimated costs retroactively, which is susceptible to error. To minimize error, we worked closely with study administrators to identify intervention resources and used gold-standard value-estimation methodology. Although retroactive resource identification and cost estimation is a common practice, prospective estimations are more reliable. Additionally, lay counselors were present during administration of baseline and follow-up assessments, which could introduce presentation bias.

Our economic evaluation is a rare contribution to the youth global mental health intervention literature and showcases how low-intensity interventions can have meaningful mental health impacts. With budgets for mental health decreasing and the need for prevention and treatment expected to increase, economic evaluations of mental health interventions are needed to guide mental health investment decisions. Sources of cost-savings included delivering the intervention in a group format at schools and training lay providers to deliver the intervention. Our results suggest that lay providers, after screening and training, can effectively deliver a mental health intervention. Of note, *Shamiri* condition was not superior to the control condition for the RCSC criteria until the 7-month follow-up. When using the RCSC criteria at 7-month follow-up, 43% (vs. 29%) of *Shamiri* students met such criteria for depression symptom reduction and 54% (vs. 42%) met the criteria for anxiety symptom reduction. This “sleeper effect” serves as a useful reminder that clinical benefits are not always apparent immediately after treatment and reiterates the importance of using longitudinal research designs in clinical studies. The effectiveness of *Shamiri* lay providers is promising for the future of global mental health, especially in light of the shortage of mental health providers with post-baccalaureate degrees in LMICs [44]. *Shamiri’s* group and school-based delivery system also makes it more scalable than the traditional one-on-one psychotherapy delivery system; we believe that *Shamiri* has the potential to increase access to mental health treatment, particularly in resource-restricted LMICs.

Purchasing power and cost of living differ between the U.S. and Kenya and contribute to differences in hourly wages and costs of goods/services. Relative to the U.S., it is between 52 to 71% less expensive to live in Kenya (Expatisan.com; Livingcost.org). This cost-of-living discrepancy between the U.S and Kenya is reflected in the different salaries for psychologists based in the U.S. versus Kenya: The U.S. psychologist was estimated to make $50/hour, whereas the psychologist in Kenya was estimated to make $3.67/hour. Additionally, the U.S. dollar goes much farther in Kenya than it does in the U.S. One could purchase $43.80 worth of goods/services in Kenya for every $1 spent in the U.S. for the same goods/services [45]. Put another way, $1 could be exchanged for 43.8 × more goods and services in Kenya compared to the U.S. Making cross-country comparisons in cost-effectiveness can be problematic if differences in economic conditions are not acknowledged [46]. To maximize generalizability, *Shamiri* should be compared to mental health interventions delivered in countries with similar purchasing power and cost of living. Economic evaluations of mental health interventions in LMICs are needed so that more accurate conclusions can be generated about cost-effectiveness.

Also of note, costs associated with transportation time alone for supervisors and lay providers accounted for nearly half (44.7%) of total costs in our base case scenario. Due to the rural geography of Kenya, supervisors and providers spent more time traveling to and from the school sites than they did delivering the intervention. When adding the out-of-pocket transit fees for both supervisors and lay providers, that proportion increased to 53.7% of base case delivery costs. Although total delivery costs for *Shamiri* were quite low, future researchers and providers would be wise to acknowledge that a significant amount of resources for in-person interventions based in LMICs with a similar geographic makeup may go to transporting providers and support personnel to intervention sites. Of note, mobile phone supervision has shown promise as a feasible, cost-saving alternative to in-person supervision for lay providers in Kenya [47].

Another strength of our study was the 7-month time horizon. To put this time horizon in perspective, 16 of 42 (38%) of economic evaluations of anxiety treatment had a time horizon of less than 7 months [48]. It is important to look at outcomes beyond treatment termination because clinical improvements are not always immediately apparent or may continue to improve over time. The relationship between costs and effectiveness over time can be difficult to quantify; however, it is essential to make this quantification interpretable to clinicians and administrators to guide implementation and dissemination decisions. Our effectiveness-cost ratios, a dimensional categorization, mirrored results from the parent RCT, which suggested that adolescents in *Shamiri* achieved greater reductions in anxiety and depression symptoms over time compared to the control [21]. In other words, *Shamiri* students experienced greater reductions in GAD-7 and PHQ-8 symptoms per dollar spent.

Categorical approaches, such as estimated incidence rates, can also illustrate the relationships between intervention costs and clinical outcomes. Although these approaches de-emphasize individual differences and, as a result, are less precise, some clinicians may find it useful to dichotomize treatment outcomes as a “success” or “failure.” We used multiple definitions of clinically significant change, which favored different conditions at different time points. For example, at treatment termination, students in the control condition fared better than students in *Shamiri* for the standard definition of change for anxiety. Experts note that pressure to academically succeed may create anxiety for adolescent Kenyans [49]. This observation was consistent with our baseline findings for the sample, which showed that youths, on average, reported anxiety in the moderate range. It could also explain why a greater proportion of students in the study skills active control condition experienced certain reductions in anxiety at termination: Learning study skills may have targeted and helped assuage their main source of anxiety.

We found that *Shamiri* was superior to control for the RCSC definition of change for depression and anxiety at 7-month follow-up. Of note, the RCSC is a more valid indicator of treatment effectiveness than the standard definition of change [37]. Because costs between the two conditions did not differ, differences in cost per NNT are driven entirely by differences in effectiveness. If two treatments are equally effective, the treatment with the lower cost per NNT could be considered more cost-effective if the cost is within a decision-maker’s willingness-to-pay threshold. A recent systematic review of nine youth mental health economic evaluations included two studies that delivered school-based interventions [4]: *Not on Tobacco *[50] targeted smoking and *Planet Health *[43] targeted disordered eating. *Not on Tobacco *[50] cost $33.63 per “treated” (i.e., quit smoking) student in 2000 USD ($52.11 per student, in 2021 USD), and 10 students were estimated to quit per 100 students treated. *Planet Health *[43] cost $46,803 in 2010 USD ($56,500 in 2021 USD) to prevent one case of bulimia. However, all of these studies were conducted in the U.S. Compared to other youth interventions, of our statistically significant findings, our highest estimated cost per “treated” student (i.e., using the RCSC criteria) is acceptable at a cost of $172.72 per treated student in the U.S., but may be above a LMIC’s willingness-to-pay threshold due to differences in purchasing power. A recent economic evaluation of the *Shamiri* digital intervention in Kenya by Wasil and colleagues [34] estimated that it cost $25-$35 per student achieving clinically meaningful change, thus reiterating the cost-saving potential of interventions delivered in a single session and without an in-person provider. Regardless of treatment status, it cost between $6.80 and $20.81 to deliver *Shamiri* to each student. This is similar to the $22.40 (2020 USD) cost per participant to provide usual care plus adjunctive depression services (psychoeducation, antidepressant pharmacotherapy, and home-based follow-up) in a primary care center in Nepal [51], which has a similar purchasing power (34.3) to Kenya (43.8) [45]. Of note, in the context of different cost methodologies and outcome metrics across studies, direct comparisons of cost-effectiveness are difficult. Given the dearth of cost data on youth mental health interventions in real-world settings, it is challenging for cost-effectiveness data to guide real-world decisions. We encourage applied clinical researchers to consider reporting delivery costs in their research. Yates’ (2020) [26] instructional guide for cost-inclusive research is a useful resource.

## Conclusions

Our study supports the effectiveness and cost-effectiveness of a school-based intervention delivered by lay providers in Kenya. Our present results on costs and effectiveness can contribute to a growing body of knowledge on the costs, effectiveness, and cost-effectiveness of treatment programs targeting depression and anxiety in youth in LMICs.

## Data Availability

Links to data and intervention materials can be found in Supplements 1 through 3 of the published parent randomized controlled trial: 10.1001/jamapsychiatry.2021.1129.

## Abbreviations

ECR: Effectiveness-Cost Ratios
LMIC: Low- and Middle-Income Countries
NNT: Number Needed to Treat
RCSC: Reliable and Clinically Significant Change
RCT: Randomized Controlled Trial
USD: United States Dollars
